# Displacement Behaviour Is Associated with Reduced Stress Levels among Men but Not Women

**DOI:** 10.1371/journal.pone.0056355

**Published:** 2013-02-14

**Authors:** Changiz Mohiyeddini, Stephanie Bauer, Stuart Semple

**Affiliations:** 1 Department of Psychology, University of Roehampton, London, United Kingdom; 2 Centre for Psychotherapy Research, University Hospital Heidelberg, Heidelberg, Germany; 3 Centre for Research in Evolutionary & Environmental Anthropology, University of Roehampton, London, United Kingdom; Maastricht University Medical Centre, The Netherlands

## Abstract

Sex differences in the ability to cope with stress may contribute to the higher prevalence of stress-related disorders among women compared to men. We recently provided evidence that displacement behaviour - activities such as scratching and face touching - represents an important strategy for coping with stressful situations: in a healthy population of men, displacement behaviour during a social stress test attenuated the relationship between anxiety experienced prior to this test, and the subsequent self-reported experience of stress. Here, we extend this work to look at physiological and cognitive (in addition to self-reported) measures of stress, and study both men and women in order to investigate whether sex moderates the link between displacement behaviour and the response to stress. In a healthy study population, we quantified displacement behaviour, heart rate and cognitive performance during the Trier Social Stress Test, and used self-report questionnaires to assess the experience of stress afterwards. Men engaged in displacement behaviour about twice as often as women, and subsequently reported lower levels of stress. Bivariate correlations revealed that for men, higher rates of displacement behaviour were associated with decreased self-reported stress, fewer mistakes in the cognitive task and a trend towards lower heart rate; no relationships between displacement behaviour and stress measures were found for women. Moreover, moderation analyses revealed that high rates of displacement behaviour were associated with lower stress levels in men but not in women, and that high displacement behaviour rates were associated with poorer cognitive performance in women, but not men. These results point to an important sex difference in coping strategies, and highlight new avenues for research into sex biases in stress-related disorders.

## Introduction

Sex differences in the experience and the impact of stress are well documented; along a number of different dimensions, women experience markedly more stress than men [1]. In addition, women are significantly more likely than men to be diagnosed with stress-related disorders such as depression or anxiety [2], and their greater experience of stress may increase risk of diseases such as cancer and cardio-vascular disease [3]. Understanding the factors underlying sex differences in stress linked conditions is a major goal for psychologists, psychiatrists and other medical practitioners [4]. In this area, there is significant interest in exploring sex differences in behavioural responses to stress, as behavioural coping may affect if - and how - stress is experienced, and could be a key protective factor against stress-related disorders [5]. In addition, behavioural indicators of stress provide particularly valid insights into underlying coping mechanisms as such indicators, in contrast to self-reported stress, are not affected by impairment of memory over time [6], [7] or by nondisclosure and reporting biases [8], [9]. The majority of studies of coping behaviour have investigated affiliative [10] or aggressive [11] responses to stressful situations. More recently, however, attention has begun to focus on the potential role in coping of ‘displacement behaviour’ – a group of activities such as scratching, face touching and lip biting that appear to have no relevance to the context in which they occur [12].

The small number of studies that have been carried out in this area have provided evidence that displacement behaviour may have an important function regulating the impacts of stressful events. Pico-Alfonso *et al.* [13] found that women who showed higher rates of displacement behaviour during a stressful interview showed a lower heart rate during the post-stressor recovery period. More recently, Mohiyeddini and Semple [14] found evidence in a population of men that displacement behaviour occurring during a social stress test attenuated the relationship between the anxiety experienced immediately prior to this test, and the subsequent experience of stress. Two related mechanisms might explain the stress regulating function of displacement behaviour. Firstly, at a proximate behavioural level, displacement behaviour may allow an individual temporarily to ‘cut-off’ attention from a threatening stimulus, and this short term diversion of attention could reduce the negative arousal associated with the stimulus [15], [16]. Secondly, at the cognitive level, an attenuation of negative arousal might affect the evaluation of the situation as stressful [17]–[20].

Although the studies by Pico-Alfonso *et al.* [13] and Mohiyeddini and Semple [14] provide evidence for a stress attenuating role of displacement behaviour, as different measures of stress were used in each and in the absence of similar studies with a mixed sex population, it is unclear whether displacement behaviour regulates stress similarly in men and women. Investigating potential sex differences in the impact of such behaviour in regulating individuals' physiological, emotional - or other - responses to stress may contribute to our understanding of the widely documented sex differences in prevalence of stress related disorders and disease [2], [3], [21]. There are several reasons to expect that sex may moderate the link between displacement behaviour and the response to stress. Firstly, it is well established that stress and emotion regulation differ between the sexes [22]–[24]. For instance, empirical evidence indicates that women more often use rumination [25], catastrophizing [26] and passive and emotion-focused coping strategies [27], [28], whereas men score higher in emotional suppression - inhibiting emotion-expressive behaviour [29] – and in positive refocusing [26]. Furthermore, there is evidence for sex differences in psychological phenomena that might be related to displacement behaviour, such as the experience and expression of emotions [30], experience of stress [31], generation of stress [32], negative affectivity [33] and temperament [34].

In the present study, we exposed healthy adult men and women to an intensely stressful social situation to explore whether sex moderates the association between displacement behaviour and the self-reported experience of stress after the stressor, and the physiological and cognitive responses during this stressful event. Studying emotional, cognitive and physiological responses to acute social stress in a healthy population can provide important insights into potential pathogenic impacts of stress [35]–[37]. This approach helps to clarify the role of psychological factors in the aetiology of psychological disorders, beyond the level achievable with epidemiological studies [38]. For example, previous research has indicated that elevated response to acute psychological stress is associated with greater beta-adrenergic activation than parasympathetic response [39], affects erythron variables such as haematocrit, mean cell and the number of red blood cells [40], [41], is predictive of a higher risk of developing essential hypertension [21] and coronary heart disease such as increased left ventricular mass [42], and is positively associated with lifetime risk of heart disease [43].

We first quantified and compared men's and women's rate of displacement behaviour during a stressor. Then, in relation to stress responses, we tested hypotheses that - in line with previous findings - women would self-report a greater experience of stress after the stressor (**H1a**) as well as showing increased physiological response (**H1b**) and lower cognitive performance during this event (**H1c**). Next, in relation to the coping function of displacement behaviour, for both sexes we tested hypotheses that displacement behaviour would be negatively correlated with the self-reported experience of stress (**H2a**), and with physiological (**H2b**) and cognitive (**H2c**) responses. Finally, we examined a moderation model to test hypotheses that sex alters the strength of the relationship between displacement behaviour and the self-reported experience of stress (**H3a**), physiological response (**H3b**) and cognitive response (**H3c**).

## Methods

### Ethics statement

The project was approved at the Department of Psychology at University of Salzburg in Austria, and was carried out in accordance with the Declaration of Helsinki principles. All participants provided written informed consent.

### Power analysis

An *a priori* power analysis conducted with G-Power [44] revealed an optimal sample size of *n* = 82 to detect an effect size of η2 = 0.6 (representing a medium-high effect size) for displacement behaviour with a power 0.90 or greater and alpha = 0.05.

### Participants

82 healthy adult volunteers (50% female) were recruited through advertisements. Using a self-report questionnaire, the following criteria were applied to exclude individuals with factors that might have an impact on displacement behaviour under stress: medical conditions (e.g. heart disease, diabetes, hypertension), any current or previous clinical psychosomatic conditions (such as migraines) or psychiatric diseases, any allergies, atopic diathesis, rheumatic diseases, recreational drug use, medication or poor sleep pattern.

#### Menstruation cycle

In their review on the effects of sex and hormonal status on the physiological response to acute psychosocial stress, Kajantie and Phillips [45] concluded that it is crucial to take the menstrual phase of subjects into account in study design in order to control the natural variation of neuroendocrinological process of menstruation and the impact of oral contraceptives on physiological and emotional responses to stress. Women in the luteal phase show similar physiological responsiveness to acute social stress (such as TSST), with levels comparable to those of men [46]–[48]. Female participants in our sample were all tested in the luteal phase of the menstrual cycle, with this being assessed from their self-report on occurrence of menstruation; none of the participants were using oral contraceptives.

Participants were instructed to relax and answer the questionnaires (see below) prior to the stress exposure. Five female and six male participants were excluded due to incomplete questionnaire data, technical issues during the recording of the heart rate or behaviour, or to their taking of medication. Data from 71 participants (87% of the initial sample) were therefore included in the analysis. Females (n = 36) were on average 24.16 years old (*SD* = 3.77; range = 18–34) and males (n = 35) were on average 25.74 years old (*SD* = 3.77; range = 20–36); 90.1% of participants were native German speakers, 73.2% of participants were students and 26.8% were employees. Age was not correlated with any of our measures (Pearson correlations: for all analyses, n = 71, *r*<0.19, *p*>0.11), and there were no differences in any of these measures between native versus non-native speakers (t-tests: for all analyses t_69_<1.81, *p*>0.07). There were also no differences between groups of different occupational status (t-tests: for all analyses *t_69_*<0.83, *p*>0.41). Therefore, these three variables were not considered any further in the analyses.

### Outline of the experimental set-up and stress paradigm

Participants were informed that their behaviour and heart rate would be recorded during a simulated job interview and a cognitive test, in order to represent a professional setting. As a psychosocial stress test, we used the Trier Social Stress Test (TSST), which has repeatedly been found to induce profound levels of stress [49]. The TSST involves giving a simulated job interview (5 min) followed by a mental arithmetic task (5 min) in front of two people (one male, one female), with both tasks being done while standing. The experiment was conducted adhering to strict experimental procedures. Each subject individually underwent the following four-phase experimental procedure, lasting around 40 min overall.

#### Adaptation phase (10 min)

heart rate recording, with the subject comfortably seated on a chair in the presence of one experimenter.

#### Baseline phase (10 min)

baseline heart rate recording at rest, immediately followed by the instruction for TSST, in the presence of the same experimenter.

#### Stress phase (10 min)

interview (5 min) followed by mental arithmetic task (5 min), with heart rate and videotape recording in the presence of two experimenters. For the arithmetic task, the participants were asked to subtract an odd number (17) from a larger number (2043), and then to keep repeating this process of subtraction. In order to render the challenge more stressful, the interviewer interrupted the participants in case of miscalculation and asked him/her to start from the initial number. This TSST situation was videotaped with a camera adjusted so that the subject's face and torso were in full view.

#### Recovery phase (10 min)

Recovery heart rate recording in the presence of one experimenter.

### Quantification of Displacement Behaviour

Displacement behaviour during the TSST was measured using a revised version of the Ethological Coding System for Interviews (ECSI). ECSI is an ethogram developed for measuring nonverbal behaviour during interviews [50]. The use of the ECSI requires video recording to allow quantification of nonverbal behaviour. Subsequently, trained observers, who are unaware of the subject's verbal reports, observe the recording and score the subject's behaviour according to the patterns listed in the ECSI. The current version of the ECSI includes 37 different behaviour patterns. The present study focuses only on the displacement scale (see Table 1). Recordings for each subject were rated independently by two trained observers, and a mean of the two raters' scores calculated and used for analysis. The recording method was one–zero sampling, a form of time sampling [51]. The recording session was divided into successive 15-s sample intervals, identified to observers by a beeper. On the instant of each sample point, the observers recorded whether or not the behaviour pattern had occurred during the preceding sample interval. Before the beginning of the study, the observers were trained in order to reach an adequate level of inter-observer reliability (i.e. a kappa coefficient of at least 0.85). The assessment of inter-observer reliability was based on a sample of 40 interviews, which did not include the ones with the subjects of this study.

**Table 1:** Ethogram of the displacement behaviour recorded in this study.

Behaviour	Definition, following Troisi [50]
Groom	The fingers are passed through the hair in a combing movement
Hand-face	Hand(s) in contact with the face
Hand-mouth	Hand(s) in contact with the mouth
Scratch	The fingernails are used to scratch part of the body, frequently the head
Yawn	The mouth opens widely, roundly and fairly slowly closing more swiftly. Mouth movement is accompanied by a deep breath and often closing of the eyes and lowering of the brows.
Fumble	Twisting and fiddling finger movements with wedding ring, handkerchief, other hand.
Twist mouth	The lips are closed, pushed forward and twisted to one side.
Lick lips	The tongue is passed over the lips.
Bite lips	One lip usually the lower is drawn into the mouth and held between the teeth.

### Self-reported Experience of Stress

Experience of stressfulness of the stress paradigm was obtained by completion immediately after the TSST, using visual analogue scales (VAS) ranging from 0 to 10 with 0 indicating no stress experienced at all. VAS require that the respondents specify their level of agreement to a statement by indicating a position along a continuous line between two end-points. Empirical evidence suggests that VAS items are more reliable and show higher content validity than discrete scales such as the Likert scale, and thus a wider range of statistical methods can be applied to these measurements [52]. After cessation of the TSST, participants were required to rate whether the stress situation was relaxing/stressful, clear/confusing, controllable/uncontrollable, energizing/exhausting, interesting/boring, pleasant/unpleasant, comfortable/embarrassing, challenging/fearful, calming/frightening. The average inter-correlations of the VAS items was 0.60 (*p*<0.001) and Cronbach's alpha was 0.92.

### Physiological response to stress (Hemodynamic measure)

Heart rate data were obtained continuously via a portable heart rate monitor (Polar system, S810; Polar, Kempele, Finland) [53]. The R–R interval was quantified as the mean of each 10-min recording period (adaption, baseline, stress task, and recovery). Using the trapezoid formula, the area under the total response curve, expressed as area under the measured time points and ground from baseline to peak response (AUCg) was calculated [54]. The computation of the area under the curve captures information that is contained in repeated measurements over time, and increases the power of the testing; it is a frequently used method in psychophysiological research to estimate changes over a specific time period. Although cuff-based measurement of blood pressure can provides data on stress response, we decided not to collect blood pressure as a pilot trial with five participants indicated that they avoided hand movements in order not to affect the measurement of blood pressure.

### Cognitive response to stress (arithmetic task performance)

In addition to the standard protocol of the TSST [49] we recorded the number of mistakes made during the mental arithmetic task, as a measure of the cognitive response to stress.

### Statistical analyses

All calculations were performed using SPSS v.20 (SPSS Inc., Chicago, IL). Data are presented as mean ± SD. In case of missing data, cases were excluded listwise. Data were tested for a normal distribution using the Kolmogorov-Smirnov test before statistical procedures were applied. Results were considered statistically significant at the *p*<0.05 level.

#### a) Sex comparisons

Between sex comparisons for scores for displacement behaviour were performed by means of Student's t-test. As nine individual displacement behaviours were compared in this way, a Bonferroni correction (adjusted critical significance level = 0.05/9 = 0.0056) was applied, to take account of multiple testing and control for Type I error.

Student's t-test was also used for between sex comparisons of the self-reported experience of stress, and physiological (heart rate) and cognitive (arithmetic task performance) responses.

#### b) Bivariate correlations

For each sex, the associations between displacement behaviour, self-reported experience of stress, physiological and cognitive responses were explored using Pearson's correlations (two-tailed).

#### c) Moderation analysis

To explore interaction effects between displacement behaviour and sex, multiple regression analyses were performed. Although there were no signs of multi-colinearity (the variance inflation factor values were well below 2.5 and tolerance statistics well above 0.2), all variables were standardised in order to equate different metrics used in measuring variables [55]. The interaction term was created as a product term (sex×displacement behaviour). Computing a hierarchical multiple regression, sex and displacement behaviour were entered first in the equation, followed by the interaction term sex×displacement behaviour. A moderator effect is indicated by a significant effect of the product term while the effects of sex and displacement behaviour are controlled.

## Results

### Sex differences in displacement behaviour and measures of stress

Women displayed significantly lower rates of displacement behaviours during the TSST than men (t_69_ = 9.20; p<0.001). During the 10 minute TSST, women displayed displacement behaviours in 15.47±2.63 of the forty 15-sec time blocks; groom was the most frequent displacement behaviour (4.83±1.26), followed by fumble (2.84±0.82), lick lips (2.13±0.79), twist mouth (1.34±0.59), bite lip (1.20±0.75), hand-face (1.18±0.79), hand-mouth (1.08±0.73), scratch (0.83±0.62), yawn (0.00±0.00). Men showed displacement behaviours in 30.84±9.05 of the forty 15-sec time blocks; bite lips was the most frequent displacement behaviour (5.92±2.24), followed by groom (4.20±1.85), hand-face (4.08±2.67), hand-mouth (3.91±2.21), scratch (3.80±1.37), lick lips (3.31±1.21), fumble (3.04±1.20), twist mouth (2.41±0.91) and yawn (0.14±0.35). For all nine individual displacement behaviours, therefore, women's mean rates were lower than those of men. Following Bonferroni adjustments to account for multiple testing, significant sex differences were found for: scratch, hand-face, hand-mouth, twist-mouth, lick-lips and bite lips (t-tests: for all analyses *t_69_*>4.83, *p*<0.001). Women also reported higher levels of self-reported experience of stress in support of **H1a**, but contrary to **H1b** and **H1c** there were no sex differences with regard to the physiological response to stress (area under the curve for heart rate) or the cognitive response (number of mistakes in the arithmetic task) (Table 2).

**Table 2:** Results of between sex comparisons of displacement behaviour, experience of stress and cognitive and physiological measures.

Variables	Male (n = 35)		Female (n = 36)		*t*	*p*
	*Mean*	*SD*	*Mean*	*SD*		
Displacement behaviour	30.84	9.66	15.47	2.63	9.20	<0.001
Experience of stress	5.17	1.48	6.91	0.96	5.89	<0.001
Number of mistakes in cognitive task	6.91	2.24	7.75	3.98	1.08	0.28
Area under the curve for heart rate	2628.29	208.36	2687.22	265.33	1.04	0.30

### Bivariate correlations of displacement behaviour and measures of stress

Table 3 displays the results of the bivariate correlation analyses for male and female participants. In men, but not women, there was support for hypotheses **H2a–c**; men's rate of displacement behaviour was significantly negatively correlated with the experience of stress (*r*_35_ = −0.438, *p* = 0.009 – **H2a**) and the number of mistakes in the cognitive task (*r*_35_ = −0.409, *p* = 0.015 – **H2c**). The negative correlation with the area under the curve for heart rate approached significance (*r*_35_ = −0.325, *p* = 0.056 – **H2b**). In addition, the experience of stress was positively correlated with the number of mistakes in the cognitive task for both men (*r*_35_ = 0.655, *p*<0.001) and women (*r*_36_ = 0.701, *p*<0.001).

**Table 3:** Results of the bivariate correlation analyses for males (n = 35, below the main diagonal) and females (n = 36, above the main diagonal).

	Displacement behaviour	Experience of stress	Number of mistakes in cognitive task	Area under the curve for heart rate
Displacement behaviour		*r* = 0.286, *p* = 0.091	*r* = 0.262, *p* = 0.122	*r* = −0.182, *p* = 0.288
Experience of stress	*r* = −0.438, *p* = 0.009		*r* = 0.701, *p*<0.001	*r* = −0.037, *p* = 0.830
Number of mistakes in cognitive task	*r* = −0.409, *p* = 0.015	*r* = 0.656, *p*<0.001		*r* = 0.005, *p* = 0.976
Area under the curve for heart rate	*r* = −0.326, *p* = 0.056	*r* = 0.222, *p* = 0.199	*r* = 0.245, *p* = 0.155	

### Moderation analyses

**Figure 1:**
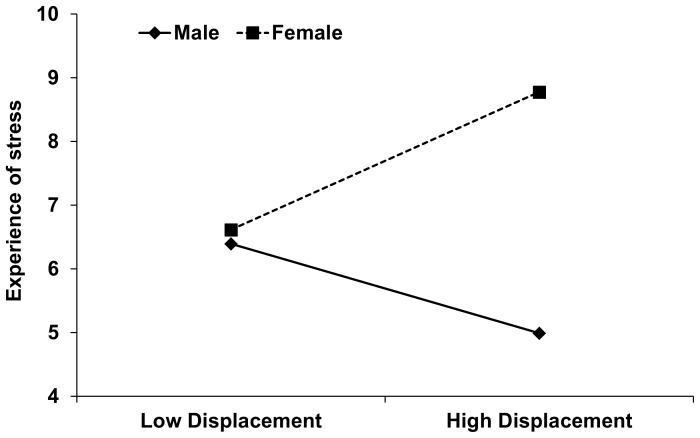
Experience of stress as a function of displacement behaviour and sex. Low displacement behaviour is defined as a score one SD or more below the mean; high displacement behaviour as one SD or more above the mean.

The results revealed a significant sex×displacement behaviour effect (*β* = 0.568, *t*_67_ = 2.507, *p* = 0.015) on the self-reported experience of stress, in support of **H3a** (see Table 4 and Figure 1). After controlling for the first order effects, the interaction between sex and displacement behaviour explained an additional 5.8% incremental variance in the experience of stress. Test of simple slopes [56] revealed that for men the experience of stress varied with displacement behaviour (*b* = −0.606, *t*_67_ = 3.274, *p* = 0.002): men with higher levels of displacement behaviour (i.e. rates higher than the mean + 1 SD) reported lower levels of experience of stress than men with lower levels of displacement behaviour (i.e. rates lower than the mean − 1 SD). By contrast, the experience of the stress was not significantly different between women with high and women with low levels of displacement behaviour (*b* = 0.934, *t*_67_ = 1.394, *p* = 0.168).

**Table 4:** Testing standardised moderator effects of sex using a hierarchical multiple regression (n = 71, * *p*<0.05. ** *p*<0.01).

Step and variables	*B*	*SE B*	95% CI	*β*	*R* ^2^	*Δ R* ^2^
**Experience of stress as dependent variable**						
Step 1					0.328	0.328*
Displacement behaviour	−0.104	0.148	−0.401, 0.192	−0.104		
Sex	0.974	0.295	0.386, 1.562	−0.491**		
Step 2					0.385	0.058*
Sex×Displacement behaviour	1.396	0.557	0.285, 2.507	0.568**		
**Number of mistakes in cognitive task as dependent variable**						
Step 1					0.033	0.033
Displacement behaviour	−0.192	0.178	−0.547, 0.163	−0.192		
Sex	−0.026	0.353	−0.731, 0.679	−0.013		
Step 2					0.107	0.074*
Sex×Displacement behaviour	1.579	0.671	0.240, 2.918	0.643*		
**Area under the curve for heart rate as dependent variable**						
Step 1					0.065	0.065
Displacement behaviour	−0.326	0.150	−0.625, −0.027	−0.380*		
Sex	−0.556	0.345	−1.245, 0.132	−0.282		
Step 2					0.069	0.004
Sex×Displacement behaviour	−0.319	0.587	−1.491, 0.853	−0.286		

**Figure 2:**
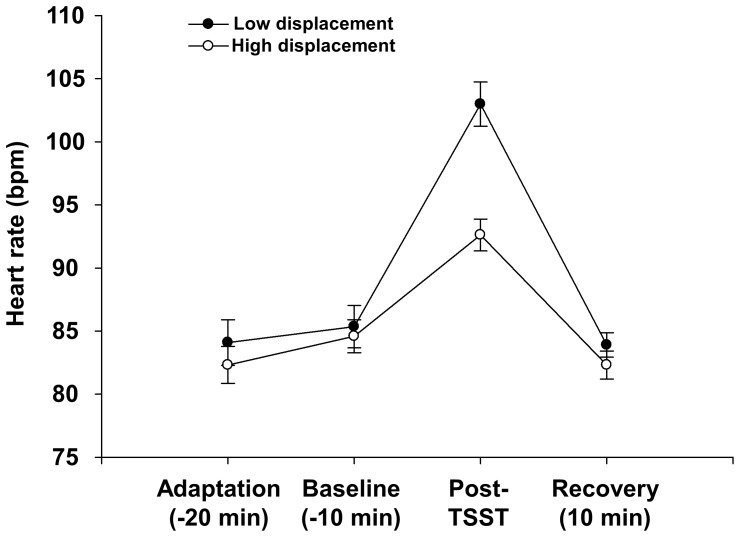
Area under the curve with respect to ground for individuals low and high in displacement behaviour. Low displacement behaviour is defined as a score one SD or more below the mean; high displacement behaviour as one SD or more above the mean.

The impact of the sex×displacement behaviour interaction on the physiological response to stress (area under the curve for heart rate) was not significant (*β* = −0.151, *t*_67_ = 0.543, *p* = 0.589) and thus **H3b** was not supported. However, the impact of displacement behaviour on the area under the curve for the whole study population was significant (*β* = −0.380, *t*_67_ = 2.173, *p* = 0.033; Figure 2). *Post-hoc* comparisons using Student's t-test with Bonferroni correction revealed that compared to individuals low in displacement behaviour (M = 102.99±10.35), individuals high in displacement behaviour (M = 92.61±7.57) showed a significantly lower heart rate in the post-stress phase (*t_69_* = 4.826, *p*<0.001)

**Figure 3:**
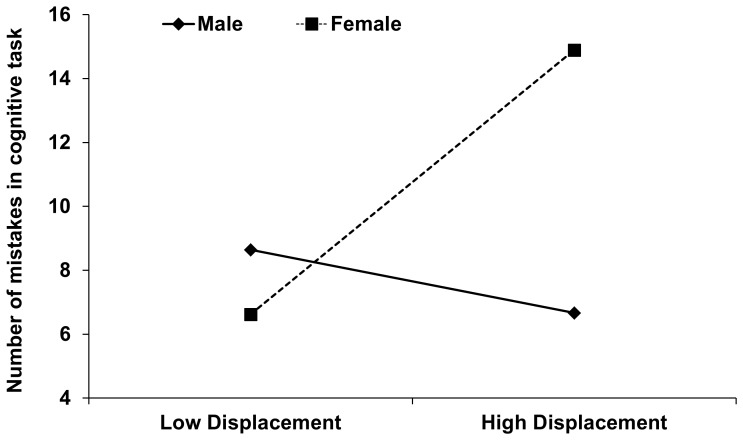
Number of mistakes in the mental arithmetic during TSST as a function of displacement behaviour and sex. Low displacement behaviour is defined as a score one SD or more below the mean; high displacement behaviour as one SD or more above the mean.

Furthermore, the sex×displacement behaviour interaction effect on the cognitive response (number of mistakes in cognitive task) was significant (*β* = 0.643, *t*_67_ = 2.354, *p* = 0.022; Table 4 and Figure 3), in support of **H3c**. After controlling for the first order effects, the interaction between sex and displacement behaviour explained an additional 7.4% incremental variance in the number of mistakes in the cognitive task. Test of simple slopes showed that women with high levels of displacement behaviour made significantly more mistakes in the cognitive task than women with low levels of displacement behaviour (*b* = 3.573, *t*_67_ = 1.97, *p* = 0.05). In men, the number of mistakes did not vary with levels of displacement behaviour (*b* = −0.853, *t*_67_ = 1.704, *p* = 0.093).

## Discussion

Studying individual responses to acute social stressors is crucial to understanding long-term pathological impacts of stress [35], [37]. In this study, we investigated the role of displacement behaviour as a coping strategy during a socially stressful situation, and tested for potential sex differences in the stress regulating function of such behaviour. During a Trier Social Stress Test (TSST), men engaged in displacement behaviour about twice as frequently as women, and self reported a lower experience of stress after the test than women; there were no sex differences in physiological (heart rate) or cognitive (performance in arithmetic task) measures of stress. Bivariate correlations revealed that for men, a higher frequency of displacement behaviour was associated with lower self-reported stress, fewer mistakes in the cognitive task and a strong trend towards a lower physiological response; among women, no such relationships were found. Moderation analyses provided evidence for important sex differences in stress regulation via displacement behaviour. Men who engaged in displacement behaviour more frequently reported a lower level of stress, while for women levels of self-reported stress did not differ depending on the frequency of displacement behaviours. In addition, women with high levels of displacement behaviour actually made more mistakes in the arithmetic task (not less, as expected); no significant association between the number of mistakes and level of displacement behaviour was observed in men. No moderating role of sex was found in relation to the physiological response to the stressor. Overall, these results point to an important sex difference in the occurrence of displacement behaviour, and in its role in coping with social stress.

To our knowledge, this is the first study that has found a significant sex difference in the rate of displacement behaviour. This may be attributable to differences between men and women in perception and interpretation of TSST as a social situation (with women focusing more on the social aspect), in how they feel about showing displacement behaviour, or in their concern at the way such behaviour may be perceived. Displacement behaviour such as scratching, licking of the lips or raising the hand to the mouth contradicts the socio-culturally determined western understanding of appropriateness [57], [58] and politeness [59] of female social behaviour and “lady-like” manners [60], [61]. Moreover, previous research indicates that women are more conscious than men about their public self [62], [63] As an internal disposition, public self-consciousness [64] denotes the awareness of oneself as a social object and reflects the tendency to think about those aspects of the self that are subject to public scrutiny and from which impressions are formed [65], [66]. In a related vein, emerging evidence from a developmental perspective highlights sex differences in the degree to which people monitor and control their behaviours and public images, to ensure that they behave appropriately [67], [68]. Females, more so than males, learn early in life that they must convey a positive image of themselves that conforms to group values of social desirability and admired traits; as a result they pay more attention to social cues, their expressive skills, and the impressions they cultivate [69]–[72].

In addition, a separate line of evidence has highlighted sex differences in self-presentation or impression management [68], which again may be expected to inhibit displacement behaviour among women. Self-presentation consists of behaviours designed to make a desired impression on others. Psychological research on sex differences in self-presentation has already revealed that women are more strongly motivated to manage their impression on others and the content of the images that they try to present, and place higher priority on creating a positive self-presentation, while men are less concerned about the image they present in social communication [73]–[78]. Accordingly, women as high self-monitors have a strong concern that their behaviour is appropriate for the social situations in which they find themselves. They are particularly sensitive to the social cues and self-presentations of others, and are thought to use such cues as guidelines for managing their own behaviour and/or creating appropriate or desirable impressions [79]. By contrast, men as low self-monitors display less concern for the situational appropriateness of their behaviour, which appears to be guided from within by dispositions, rather than by situational specifications of appropriate behaviour [80].

A number of previous studies have explored sex differences in the frequency of displacement behaviour, but found none [16], [81]–[85]. The discrepancy between the findings of these studies and our own may be due to statistical power, the context where displacement behaviour is quantified, or the magnitude or nature of the stress involved. Two of these earlier studies used a small sample in which sex differences may not have been easily detectable [16], or were carried out in contexts where displacement behaviour may be hard to express, namely during written exams [81]. The remainder of the studies were conducted in clinical settings, using interview techniques that are specifically designed to reduce feelings of stress [82]–[85]; it is possible that marked sex differences in displacement behaviour may only become apparent in intensely stressful social situations, as the TSST is designed to create.

We found no evidence that displacement behaviour alleviates stress in women; by marked contrast, displacement behaviour was associated with reduced stress in men. The latter result is in line with our previous findings that displacement behaviour regulates the self-reported experience of stress in men [14], but additionally provides evidence for the first time of effects of displacement behaviour on men's cognitive and physiological responses to stress. Overall, our findings suggest that displacement behaviour has an important function in regulating men's stress levels during challenging social situations, but that such a role in stress regulation is absent among women. The current study does not allow us to identify the exact mechanisms involved in this apparent sex difference in coping, but as a significant proportion of the stress that people experience derives from social situations [30], women's failure to regulate stress via displacement behaviour in these contexts may potentially contribute to their higher prevalence of stress-related disorders. Further studies explicitly exploring the link between displacement behaviour during non-social stressful situations (e.g. where participants have to perform under time pressure without the presence of an audience) would provide further insight into this issue.

The finding in our moderation analyses that women who showed high levels of displacement behaviour during the TSST made more mistakes in the challenging arithmetic task is unexpected. One possible explanation for this result is that engaging in displacement behaviour impairs cognitive performance; displacement behaviour has been proposed to ‘cut-off’ attention temporarily from a stressful or threatening stimulus [15], and this short-term diversion of attention could reduce the ability to deal with a mentally challenging task. Alternatively, women who made more mistakes in the cognitive task may have perceived the situation as more stressful [86], which in turn increased their displacement behaviour. Finally, it is possible that awareness of their own engagement in displacement behaviour - and, for example, the impression this may give - elevates stress levels among women [87], [88], and this in turn impairs performance in the cognitive task. The lack of a significant interaction between sex and displacement behaviour in the association with our physiological stress measure (heart rate) contrasts with the results in relation to both the self-reported experience and cognitive measure of stress. Together, these results may indicate that the pathways by which sex and displacement behaviour are linked with cardiovascular activity are different to the mechanisms by which sex and displacement behaviour interact to moderate the experience of stress and cognitive stress measure in this sample.

Strengths of the present study include the rigorous experimental procedure, as well as the integration of ethological observation with multiple stress assessment methods. A key limitation of this work, however, is that the laboratory based experimental paradigm has low ecological validity. In addition, the moderate sample size means that some of the observed associations - particularly in moderation analyses - may have failed to reach statistical significance due to a lack of power. The present study sample was also relatively young, and as age can affect both the experience and impact of stress [1], [89], [90], caution should be exercised if extrapolating findings to older age cohorts. Finally, our assessment schedule meant it was not possible to draw firm conclusions about causal relationships between the frequency of displacement behaviour and our stress measures. Further research into displacement behaviour in the context of stressful situations promises to enhance our understanding of such behaviour as a coping strategy, and how this role may differ between the sexes. Future studies should explore other coping responses in addition to displacement behaviour, in order to determine the potentially unique contribution of displacement behaviour within the spectrum of strategies deployed by men and by women to cope with stressful situations.
